# Delineating the short- and long-term impact of ionizing radiation on antigen-inexperienced CD8^+^ T cell subsets

**DOI:** 10.1172/jci.insight.194201

**Published:** 2025-08-05

**Authors:** Mohammad Heidarian, Shravan K. Kannan, Whitney Swanson, Thomas S. Griffith, John T. Harty, Vladimir P. Badovinac

**Affiliations:** 1 Department of Pathology,; 2 Experimental Pathology Graduate Program, and; 3 Interdisciplinary Graduate Program in Immunology, University of Iowa, Iowa City, Iowa, USA.; 4 Department of Urology and; 5 Center for Immunology, University of Minnesota, Minneapolis, Minnesota, USA.; 6 Minneapolis VA Health Care System, Minneapolis, Minnesota, USA.

**Keywords:** Immunology, Inflammation, Radiation therapy, T cells

## Abstract

Radiation-induced lymphopenia (RIL) remains a challenging side effect of radiation therapy that is often associated with poor prognosis and reduced overall survival. Although CD8^+^ T cells are highly radiosensitive, the dynamics of quantitative and qualitative changes to the CD8^+^ T cell pool following exposure to high doses of ionizing radiation (IR) remain understudied. Herein, we sought to determine the long-term impact of sublethal whole body irradiation (WBI) on the antigen-inexperienced (Ag-inexperienced) CD8^+^ T cell pool, comprising naive (T_N_) and virtual memory (T_VM_) CD8^+^ T cells. We show that although both T_N_ and T_VM_ cells gradually regenerated after WBI-induced loss, T_N_ recovery occurred only through de novo thymic production. Despite the numerical restoration, the subset and phenotypic composition of postrecovery Ag-inexperienced CD8^+^ T cells did not qualitatively recapitulate the pre-WBI state. Specifically, the frequency of T_VM_ cells is increased, especially during the early stages of recovery. Within the T_N_ subset, a lasting overrepresentation of Ly6C^+^CD122^+^ cells and an altered TCR clonotype diversity are also observed. Overall, our data highlight the dynamic changes to the Ag-inexperienced CD8^+^ T cell pool upon recovery from RIL

## Abstract 2

Antigen-inexperienced CD8+ T cells undergo lasting phenotypic and subset composition change following recovery from radiation-induced lymphopenia.

## Introduction

Radiotherapy is a common approach used in more than 50% of solid tumor treatment plans. Ionizing radiation (IR) can directly kill tumor cells through induction of DNA damage and act as an “in situ vaccine” to trigger antitumor immune response (1–3). IR can also cause deleterious side effects on healthy tissues (4–6). For example, lymphocytes are one of the most radiosensitive cells among the immune system. Although cells of lymphoid origin differ in their susceptibility to IR-induced cell death, with B cells being the most radiosensitive, followed by T and NK cells, even low doses of IR (as little as 0.5 Gy) can rapidly induce lymphocyte death (7–9). In fact, more than 40% of patients with solid tumors receiving radiotherapy as part of their treatment regimen develop severe lymphopenia (10), which is associated with poor prognosis and reduced overall survival (11, 12). In addition to increased susceptibility to both previously and newly encountered pathogens, prolonged radiation-induced lymphopenia (RIL) has been associated with decreased efficacy of immunotherapy approaches (13, 14). While treatment plans with minimal unwanted IR exposure to lymphocyte-rich niches have been proposed to prevent RIL (15, 16), further understanding of the factors that contribute to qualitative and quantitative recovery of lymphocytes paves the path to a more efficient treatment paradigm.

CD8^+^ T cells are potent mediators of immunity against intracellular pathogens and malignancies (17–19). At steady state, naive CD8^+^ T (T_N_) cells remain quiescent, but cognate antigen (Ag) encounter along with costimulatory and inflammatory signals trigger massive proliferation and differentiation of T_N_ cells to activated effector CD8^+^ T cells that combat the infected/cancerous cells and eventually give rise to long-lasting bona fide memory CD8^+^ T (T_MEM_) cells (20–23). While continuous TCR contact with self-antigens and homeostatic cytokines are required for the long-term survival of T_N_ cells (24–26), these signals can also mediate the transition of T_N_ cells to the semidifferentiated virtual memory CD8^+^ T (T_VM_) cells that share phenotypic similarities with T_MEM_ cells. T_VM_ cells generally represent a smaller subset of Ag-inexperienced CD8^+^ T cells than T_N_ cells (27–29). Maintaining a numerically stable and heterogeneous population of Ag-experienced and -inexperienced CD8^+^ T cells with a highly diverse repertoire of unique TCRs is crucial in conferring protection against a wide range of Ags (30–33).

Thymic production and homeostatic proliferation (HP) of peripheral T cells are 2 mechanisms that can regenerate the T cell compartment under lymphopenic conditions (34, 35). Owing to the higher capacity of CD8^+^ T cells to undergo HP than CD4^+^ T cells, numerical recovery of CD8^+^ T cells is less dependent on thymic output (36, 37). The capacity of CD8^+^ T cells to undergo HP also does not decline with age, unlike the thymic output, making HP the major regenerative mechanism for CD8^+^ T cells in adults (36, 38). Although HP may reestablish the number of CD8^+^ T cells, it can result in preferential recovery of certain TCR clones and result in altered clonal diversity of the CD8^+^ T cell compartment, highlighting the importance of de novo thymic emigrants to balance CD8^+^ T cell recovery (39, 40). Here, we investigated the kinetics of recovery and composition of the Ag-inexperienced CD8^+^ T cells following sublethal whole body irradiation (WBI). We also examined the contribution of newly generated T cells from thymus and HP of IR-experienced CD8^+^ T cells to the recovery of Ag-inexperienced CD8^+^ T cell pool. Our results suggest that unlike bona fide T_MEM_ cells, Ag-inexperienced CD8^+^ T cells were gradually restored. In the absence of a thymus, T_N_ recovery was severely compromised, and CD8^+^ T cells that survived the IR exposure only gave rise to T_VM_ cells. Euthymic mice, on the other hand, were able to regenerate both subsets of Ag-inexperienced CD8^+^ T cells, with the postrecovery T_N_ cells appearing to be phenotypically and clonally distinct. These data collectively suggest that exposure to high doses of IR can have a profound, long-term impact on the composition of Ag-inexperienced CD8^+^ T cells.

## Results

### Ag-inexperienced CD8^+^ T cells are rapidly lost but gradually restored following sublethal WBI.

**Figure 1:**
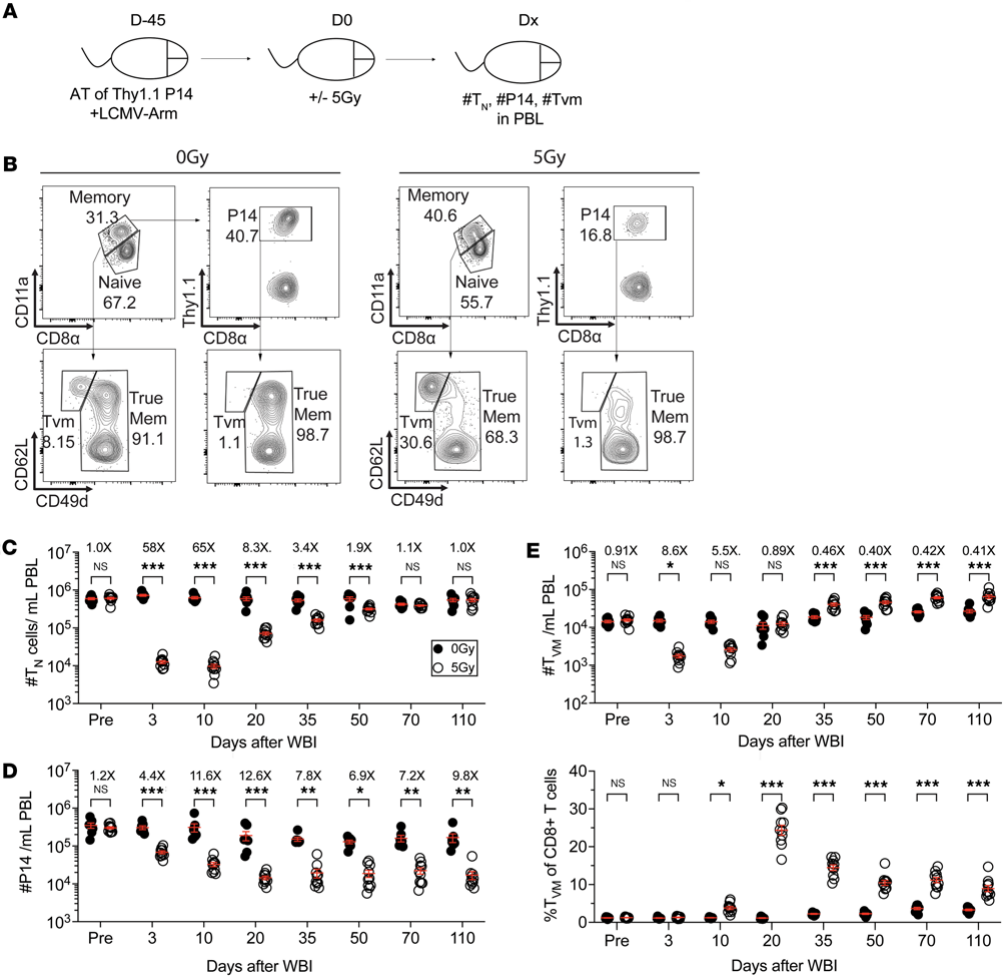
Numbers of Ag-inexperienced and memory CD8^+^ T cells are differentially altered following WBI. (**A**) Experimental design: 10^4^ naive Thy1.1^+^ P14 CD8^+^ T cells were adoptively transferred into Thy1.2^+^ naive hosts, followed by LCMV-Armstrong infection to generate memory P14 CD8^+^ T cells. At 45 days later, the memory P14 chimeric mice were exposed to mock (0 Gy) or 5 Gy dose of WBI. Analysis was performed on the indicated time points after irradiation from peripheral blood lymphocytes (PBL). Dx, days after WBI. (**B**) Representative flow plots of naive (T_N_) and virtual memory (T_VM_) CD8^+^ T cells and memory P14 cells from D20 after WBI 0 Gy and 5 Gy mice. Numbers of (**C**) T_N_ cells, (**D**) memory P14 cells, and (**E** top panel) T_VM_ cells were assessed at indicated time points in PBL analysis. (**E** bottom panel) Frequency of T_VM_ cells of total CD8^+^ T cells was also determined. Data in **A**–**E** are representative of 2 independent experiments with *n* = 5–10 mice per group in each experiment. Statistical significance was determined by 2-way ANOVA with Bonferroni’s multiple comparisons post hoc test using GraphPad Prism. Graphs show the mean ± SEM with each symbol representing 1 mouse. Individual *P* values are noted on respective graphs or are summarized as follows: *P < 0.05, **P < 0.01, ***P < 0.001.

Bona fide T_MEM_ cells undergo a permanent attrition following exposure to a 5 Gy dose of WBI (41). To compare the kinetic of loss and recovery of Ag-inexperienced CD8^+^ T cells and bona fide T_MEM_ cells that are cognate Ag-experienced cells, we exposed lymphocytic choriomeningitis virus–experienced (LCMV-experienced) P14 chimeric mice to either mock or sublethal WBI (Figure 1A). To enumerate T_N_ cells, we used a surrogate marker approach by which T_N_ cells can be distinguished from CD8^+^ T cells with Ag-experienced/memory phenotype based on higher expression of CD8α and lower expression of CD11a, a component of integrin molecule lymphocyte function-associated antigen 1(42, 43). T_N_ cells demonstrated a tissue-wide and dose-dependent depletion as early as 1 day after exposure to different doses of WBI (Supplemental Figure 1; supplemental material available online with this article; https://doi.org/10.1172/jci.insight.194201DS1). Consistent with the previous results (41), the number of T_N_ cells, but not memory P14 cells, started to increase between day 10 (D10) and D20 after WBI and steadily rose to repopulate the T_N_ compartment (Figure 1, B–D). We also investigated the numerical changes to T_VM_ T cells in the same hosts following WBI. T_VM_ cells are Ag-inexperienced CD8^+^ T cells that express many markers found on bona fide T_MEM_ cells despite lacking prior Ag exposure (27). CD49d, an integrin upregulated only on effector and Ag-experienced memory CD8^+^ T cells, is a key marker used to distinguish T_VM_ cells from true Ag-experienced and T_MEM_ cells (44–46). Despite sharing the memory phenotype with bona fide T_MEM_ cells, T_VM_ cells followed a similar pattern of gradual numerical recovery as the T_N_ cells. In fact, the T_VM_ cells appeared to be overrepresented in irradiated hosts for extended periods after WBI (Figure 1E). These findings indicated that unlike bona fide T_MEM_ cells, the Ag-inexperienced CD8^+^ T compartment, namely T_N_ and T_VM_ cells, numerically recover following WBI.

**Figure 2:**
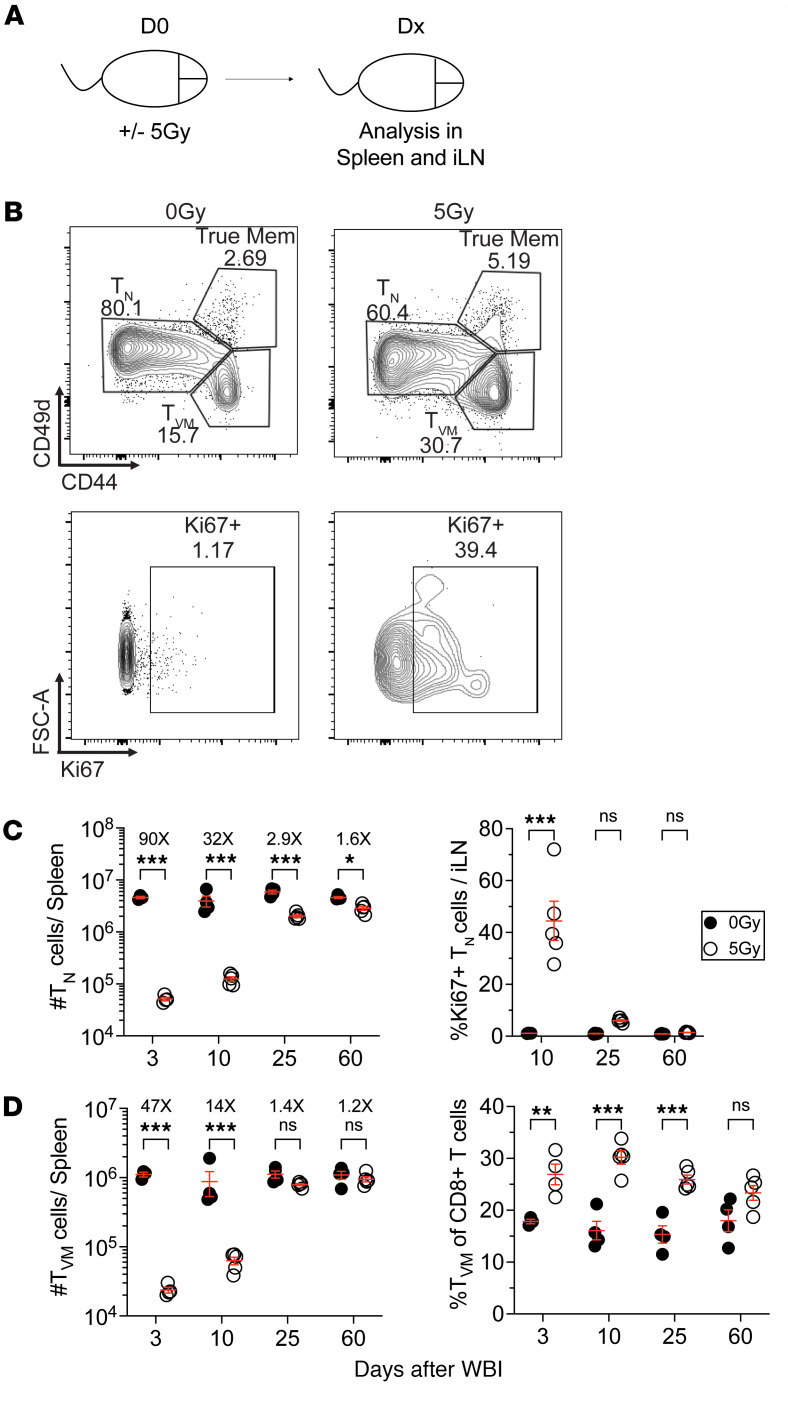
Naive and virtual memory CD8^+^ T cells follow similar kinetic of loss and recovery following WBI. (**A**) Experimental design: specific pathogen–free (SPF) mice were subjected to either 0 Gy or 5 Gy dose of WBI. At indicated time points, mice were euthanized for cellular analysis. iLN, inguinal lymph node. (**B**) Representative flow plots of naive (T_N_), virtual memory (T_VM_), and true memory CD8^+^ T cells and Ki67 expression by T_N_ cells in 0 Gy and 5 Gy mice at D25 after WBI. FSC, forward scatter. (**C**) Number of T_N_ cells per spleen (left) and frequency of Ki67^+^ T_N_ cells in inguinal lymph node at indicated time points. (**D**) Number of T_VM_ cells (left) and frequency of T_VM_ cells of total CD8^+^ T cells per spleen at indicated time points. Data in **A**–**D** are representative of 2 independent experiments with *n* = 4–5 mice per group at each time point. Statistical significance was determined by 2-way ANOVA with Bonferroni’s multiple comparisons post hoc test using GraphPad Prism at each time point. Graphs show the mean ± SEM with each symbol representing 1 mouse. Individual *P* values are noted on respective graphs or are summarized as follows: *P < 0.05, **P < 0.01, ***P < 0.001.

We next investigated the loss and recovery of Ag-inexperienced CD8^+^ T cells in specific pathogen–free (SPF) mice following WBI (Figure 2A). To define T_N_ and T_VM_ cells, we used a different gating strategy where T_N_ (CD44^lo^) and T_VM_ (CD44^hi^CD49d^lo^) were distinguished from true Ag-experienced (CD44^hi^CD49d^hi^) CD8^+^ T cells in SPF mice (Figure 2B). Following a WBI-mediated loss of T_N_ cells, vigorous proliferation of T_N_ cells ensued, as demonstrated by a substantial increase in the expression of Ki67 in T_N_ cells, leading to a substantial numerical recovery of T_N_ cells between D10 and D25 after WBI and ultimately returning to the baseline values by D60 (Figure 2C). T_VM_ cells also demonstrated a similar pattern of loss and recovery as T_N_ cells; however, the overrepresentation of T_VM_ was only observed until the T_N_ pool was fully reconstituted (Figure 2D). These data collectively suggest that T_N_ and T_VM_ cells, despite their phenotypic differences, follow a similar numerical drop and recovery following WBI in hosts with pathogen a history of pathogen exposure and SPF hosts.

### Recovery of T_N_ cells requires de novo thymic output.

**Figure 3:**
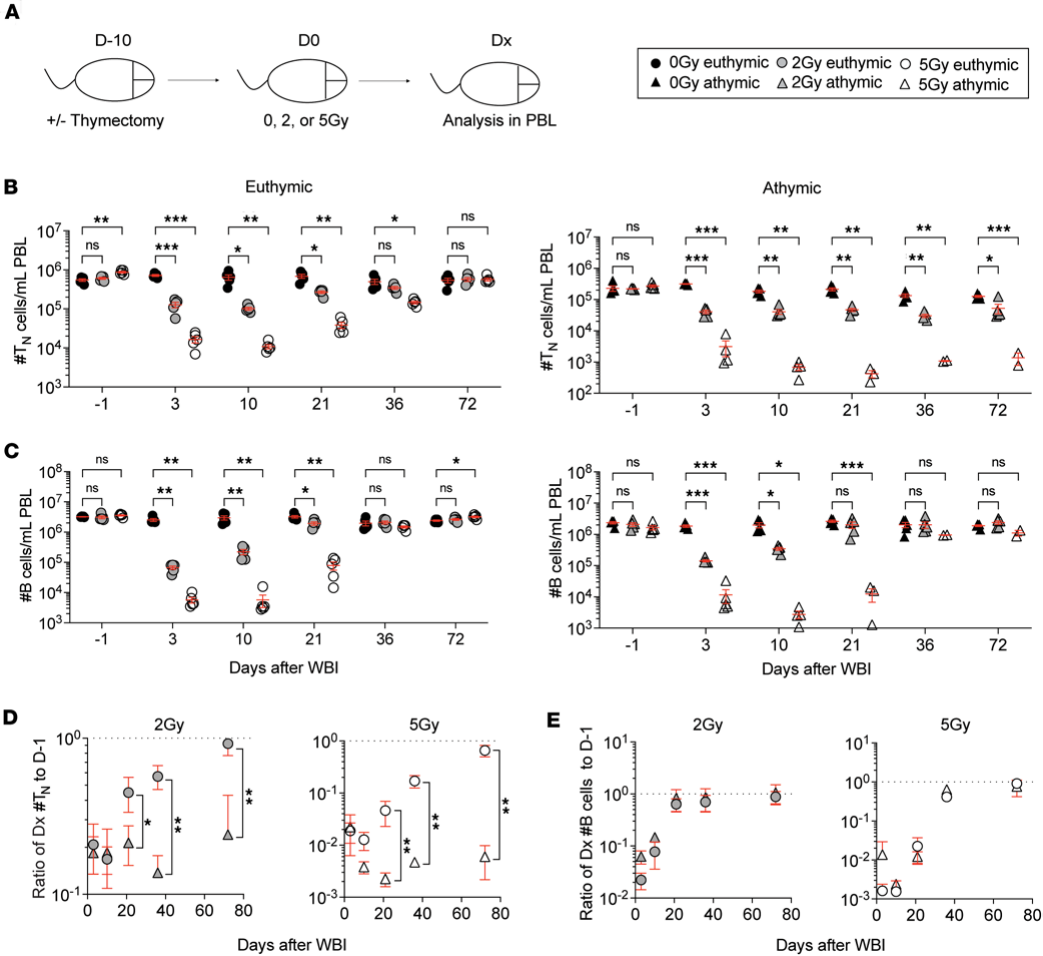
Thymus is required for naive CD8^+^ T cell numerical recovery. (**A**) Experimental design: thymectomized and euthymic SPF mice were subjected to 0 Gy, 2 Gy, or 5 Gy dose of WBI and cellular analysis was performed in PBL longitudinally. (**B**) Number of naive (T_N_) CD8^+^ T cells in blood of euthymic (left) and athymic mice (right) at indicated time points. (**C**) Number of CD19^+^ B cells in blood of euthymic (left) and athymic mice (right) at indicated time points. Number of T_N_ cells (**D**) and B cells (**E**) at each indicated time point was divided to the pre-WBI (D-1) number from the same mouse, and the mean of this ratio was plotted for 2 Gy (left) and 5 Gy (right) euthymic and athymic mice. Data in **A**–**E** are representative of 2 independent experiments with *n* = 2–5 mice per group in each experiment. Statistical significance was determined by 2-way ANOVA with Bonferroni’s multiple comparisons post hoc test. Graphs in **B** and **C** show the mean ± SEM, with each symbol representing 1 mouse. Graphs in **D** and **E** show the mean ± SD. Individual *P* values are noted on respective graphs or are summarized as follows: *P < 0.05, **P < 0.01, ***P < 0.001.

We next sought to determine the in vivo origin of the emerging T_N_ cells that gradually replenish after WBI. Specifically, we asked the question whether the T_N_ cells that survived the radiation exposure had the capacity to repopulate the T_N_ compartment. To this end, we obtained thymectomized mice that had their thymi removed at 6 weeks of age. These athymic mice allowed us to examine the impact of IR-exposed T_N_ cells versus new thymic emigrants on the numerical recovery of T_N_ cells after WBI. The euthymic and athymic mice were exposed to mock (0 Gy), 2 Gy, and 5 Gy doses of WBI, and the number of T_N_ cells and B cells were longitudinally monitored in the PBL (Figure 3A). It was noted that the 5 Gy dose of WBI was no longer sublethal for the athymic mice, as more than 50% of the mice had died by D36 after WBI. Consistent with previous results, T_N_ cells experienced a dose-dependent numerical decline followed by a gradual recovery in euthymic mice (Figure 3, B and D). However, the number of T_N_ cells in athymic mice stayed low for more than 2 months and never returned to the pre-WBI levels. The sustained loss was specific to T_N_ cells as B cells from both euthymic and athymic mice demonstrated similar kinetics of numerical loss and recovery after WBI (Figure 3, C and E). These data suggest the peripheral T_N_ cells that survive IR exposure cannot adequately repopulate the T_N_ compartment after WBI.

Previous results highlighted how the T_N_ subset repopulation was impaired in the absence of thymic emigrants after WBI. If the T_N_ subset recovery was mainly mediated by after WBI thymic emigrants, thymic reconstitution should occur prior to numerical recovery of T_N_ cells. Hence, we next investigated the kinetic of loss and recovery of thymocytes following WBI (Supplemental Figure 2A). In agreement with previous reports (47), thymocytes were rapidly lost following a 5 Gy dose of WBI, with the double-positive (DP) thymocytes being the most radiation-sensitive subset and effectively abrogated at D3 after WBI while other subsets were numerically unaltered at this time point. Interestingly, DP cells from 5 Gy hosts were rapidly restored by D10 and demonstrated similar fold change to 0 Gy controls as double-negative, single-positive CD4, and single-positive CD8 thymocytes until full recovery was observed by D60 (Supplemental Figure 2, B–D). The substantial increase in thymocyte numbers occurred between D3 and D10 after WBI, which preceded the marked increase in T_N_ numbers, observed after D10 after WBI. These data indicate that the recovery of T_N_ cells does not occur until the number of thymocytes is sufficiently restored following WBI.

**Figure 4:**
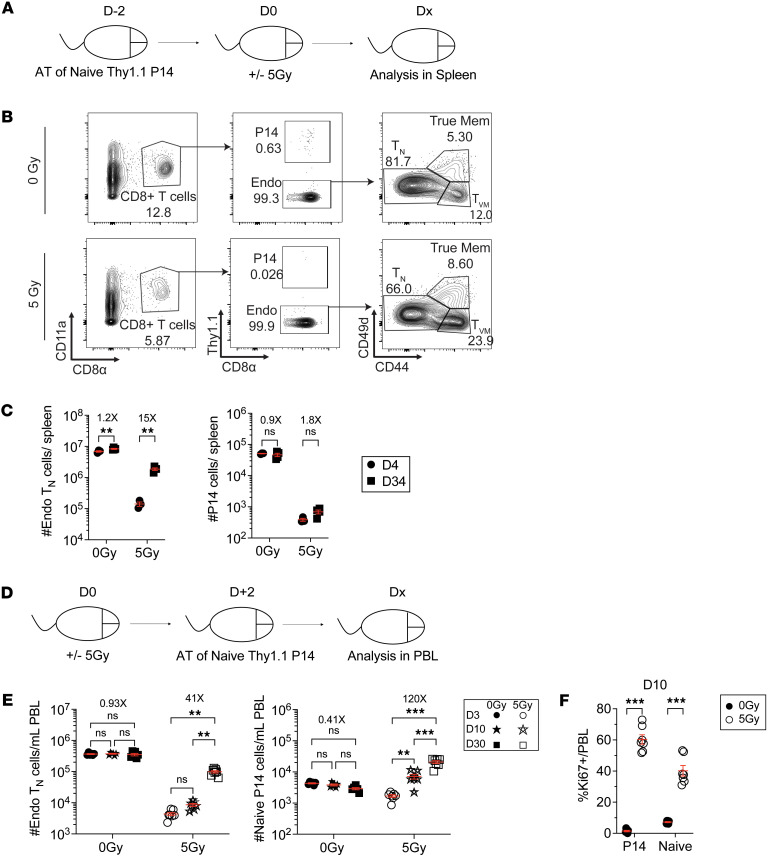
WBI-surviving naive CD8^+^ T cells exhibit diminished proliferative capacity. (**A**) Experimental design: 10^6^ naive Thy1.1^+^ P14 CD8^+^ T cells were adoptively transferred into Thy1.2^+^ naive hosts followed by exposure to 0 Gy or 5 Gy dose of WBI 2 days later. Spleens were harvested and analyzed at indicated time points. (**B**) Representative flow plots of P14 CD8^+^ T cells and endogenous naive (T_N_) and virtual memory (T_VM_) CD8^+^ T cells in 0 Gy and 5 Gy mice at D34 after WBI. (**C**) Number of endogenous T_N_ cells (left) and P14 cells (right) in 0 Gy and 5 Gy hosts at indicated time points after WBI. (**D**) Experimental design: 7.5 × 10^5^ naive P14 CD8^+^ T cells were adoptively transferred to Thy1.2^+^ naive hosts that had undergone 0 Gy or 5 Gy WBI 2 days prior. Longitudinal blood analysis was performed at indicated time points. (**E**) Number of endogenous T_N_ cells (left) and naive P14 cells (right) in 0 Gy and 5 Gy hosts at indicated time points after WBI. (**F**) Frequency of P14 and T_N_ cells that express Ki67 in 0 Gy and 5 Gy hosts at D10 after WBI. Data in **A**–**F** are representative of 2 independent experiments with *n* = 5–8 mice per group in each experiment. Statistical significance was determined by 2-way ANOVA with Bonferroni’s multiple comparisons post hoc test using GraphPad Prism. Graphs show the mean ± SEM with each symbol representing 1 mouse. Individual *P* values are noted on respective graphs or are summarized as follows: **P < 0.01, ***P < 0.001.

Lymphopenia can induce robust proliferation of CD8^+^ T cells through HP (48). While IR can induce T_N_ cell death either directly through irreversible DNA damage or indirectly via heightened inflammatory signals and a plethora of cytokines released in response to IR-induced cell death (49, 50), little is known about the fate of the TN cells that survive the initial IR exposure and early IR-driven inflammation. We next asked whether exposure to IR or early IR-mediated inflammation affected the ability of T_N_ cells that survive high doses of IR to undergo HP following RIL. We adoptively transferred Thy1.1^+^ naive P14 cells, which cannot be de novo–generated by thymopoiesis, to Thy1.2^+^ hosts, followed by 0 or 5 Gy WBI 2 days later, and assessed the number of endogenous T_N_ and P14 cells at D4 and D34 after WBI (Figure 4, A and B). As expected, the number of P14 and endogenous T_N_ cells did not change between D4 and D34 in 0 Gy hosts. While a significant 15-fold increase in the number of endogenous T_N_ cells occurred in this 30-day period, the number of P14 cells, as a surrogate for IR-experienced T_N_ cells, remained low (Figure 4C). Conversely, when Thy1.1^+^ naive P14 cells were adoptively transferred once there was an established RIL but P14 cells were not directly exposed to IR (Figure 4D), the number of naive P14 cells increased over time through vigorous cell proliferation similar to the endogenous T_N_ cells (Figure 4, E and F). These data collectively demonstrate that the direct exposure to high dose of IR or the early IR-induced inflammation following WBI impaired the capacity of IR-experienced T_N_ cells to respond to homeostatic cues as the environment after WBI is permissive for proliferation of radiation-inexperienced T_N_ cells.

### After recovery T_N_ cells display altered phenotypic and clonal composition.

**Figure 5:**
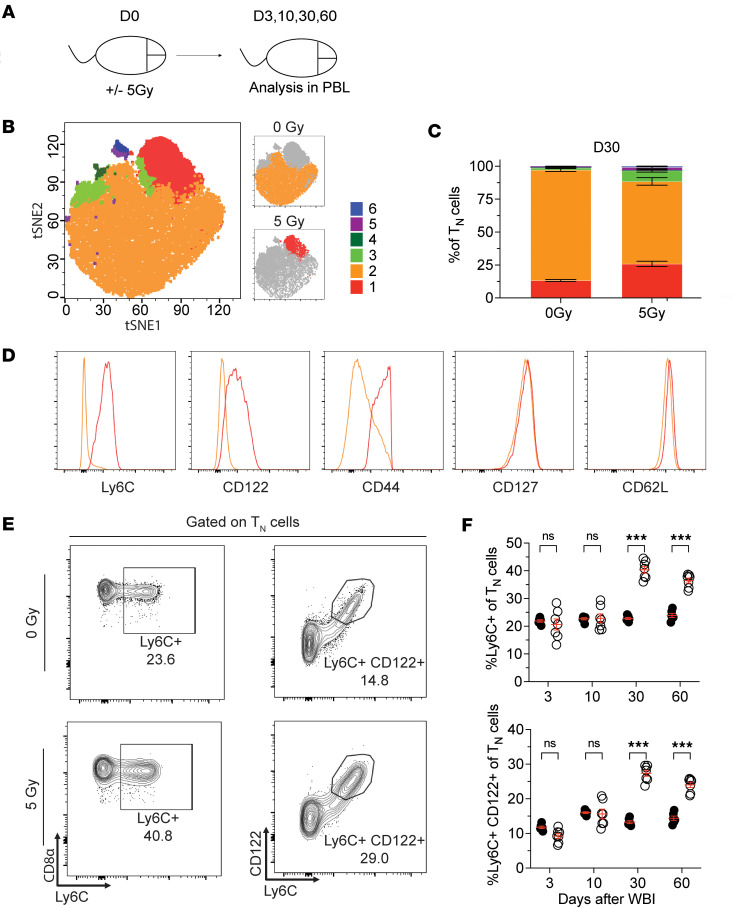
Phenotypic composition of T_N_ cells undergoes lasting alterations following numerical recovery from WBI. (**A**) Experimental design: SPF mice were subjected to 0 Gy or 5 Gy dose of WBI, and cellular analysis was performed in PBL longitudinally. (**B**) Left: t-SNE analysis of PBL displaying FlowSOM defined clusters among D30 naive (T_N_: CD44^lo^CD8α^+^) CD8^+^ T cells based on expression of CD8α, CD11a, CD44, CD62L, CD49d, Ly6C, CD127, CD122. Right: clusters that are most robustly enriched in 0 Gy (top) and 5 Gy (bottom) groups. (**C**) Cluster distribution among T_N_ cells of 0 Gy and 5 Gy mice. (**D**) Histograms of Ly6C, CD122, CD44, CD127, and CD62L surface expression comparing clusters 1 and 2. (**E**) Representative flow plots and (**F**) frequency of Ly6C^+^ and Ly6C^+^CD122^+^ T_N_ cells at indicated time points after WBI. Data in **A**–**F** are representative of 2 independent experiments with *n* = 5–7 mice per group in each experiment. Statistical significance was determined by 2-way ANOVA with Bonferroni’s multiple comparisons post hoc test using GraphPad Prism. Graphs show the mean ± SEM with each symbol representing 1 mouse. Individual *P* values are noted on respective graphs or are summarized as follows: ***P < 0.001.

Thus far we have investigated the numerical changes to T_N_ cells following WBI and the factors that influence the efficacy of the after WBI T_N_ reconstitution. We next turned our focus to assessing the qualitative changes that may also occur to the composition of T_N_ cells following recovery from WBI. Recent studies have demonstrated the impact of homeostatic cues in shaping the phenotypic and functional heterogeneity of the T_N_ population. Specifically, Ly6C expression demarcates a subpopulation of T_N_ cells with enhanced cognate Ag-mediated effector function and Ag-independent bystander responses (51–53). We sought to define any potential changes to the phenotype of T_N_ cells throughout the recovery process after WBI (Figure 5A). D30 T_N_ cells were clustered based on expression of Ly6C, CD122, CD44, CD62L, CD127, CD11a, and CD49d using FlowSOM, and these clusters were projected into t-distributed stochastic neighbor embedding (t-SNE) plots for further analysis (Figure 5B). Although clusters 1 and 2 together accounted for the majority of the naive CD8^+^ T cells in both groups, cluster 1 was more enriched within 5 Gy T_N_ cells whereas cluster 2 was more abundant within 0 Gy T_N_ cells (Figure 5, B and C). Both clusters similarly expressed CD127 and CD62L, 2 canonical markers of T_N_ cells; however, Ly6C and CD122 expression was significantly higher in cells within cluster 2, suggesting the representation of Ly6C^+^CD122^+^ T_N_ cells was increased in 5 Gy mice (Figure 5D). Our longitudinal PBL analysis of the frequency of Ly6C^+^ and Ly6C^+^CD122^+^ T_N_ cells revealed the enrichment of these subpopulations lasts up to D60 after WBI and was not due to preferential post-WBI survival (Figure 5, E and F). These findings showed the post-WBI T_N_ subset undergoes lasting phenotypic alterations.

Given the propensity of T_N_ cells with higher affinity for self-peptide–MHC ligands to undergo HP following RIL than low-affinity T_N_ cells, we next investigated to what extent the clonal diversity of the T_N_ subset changes once numerical recovery is achieved. To this end, TCRβ variable chain isotype frequencies within the T_N_ population of 0 Gy and 5 Gy mice were compared early (D3) and late (D60) after WBI (Supplemental Figure 3, A and B). Importantly, the TCR Vβ profile of 0 Gy splenic T_N_ cells examined at 4 time points indicated a very stable pool with little to no change to the “status quo” T_N_ diversity within this period (Supplemental Figure 3C). This assessment enabled us to determine whether T_N_ cells expressing distinct Vβ chains were differentially lost (D3) or recovered (D60) following WBI. While the frequency of cells expressing Vβ3, Vβ5.1/5.2, Vβ6, and Vβ10b did not change after the number of T_N_ cells acutely reduced, Vβ6 and Vβ5.1/5.2 cells were differentially recovered, leading to the underrepresentation of the former and overrepresentation of the latter population at D60 after WBI. Additionally, T_N_ cells expressing Vβ2, Vβ8.1 /8.2, Vβ8.3, Vβ11, Vβ13, and Vβ14 were all differentially susceptible to WBI-induced loss, and the frequency of Vβ8.1/8.2 and Vβ13 expressing cells still remained lower than 0 Gy counterparts 60 days after WBI (Supplemental Figure 3D). These data collectively indicated the altered clonal diversity within the T_N_ repertoire after recovery from WBI.

### Both newly emerging and radiation-survived naive CD8^+^ T cells can differentiate into T_VM_ cells.

**Figure 6:**
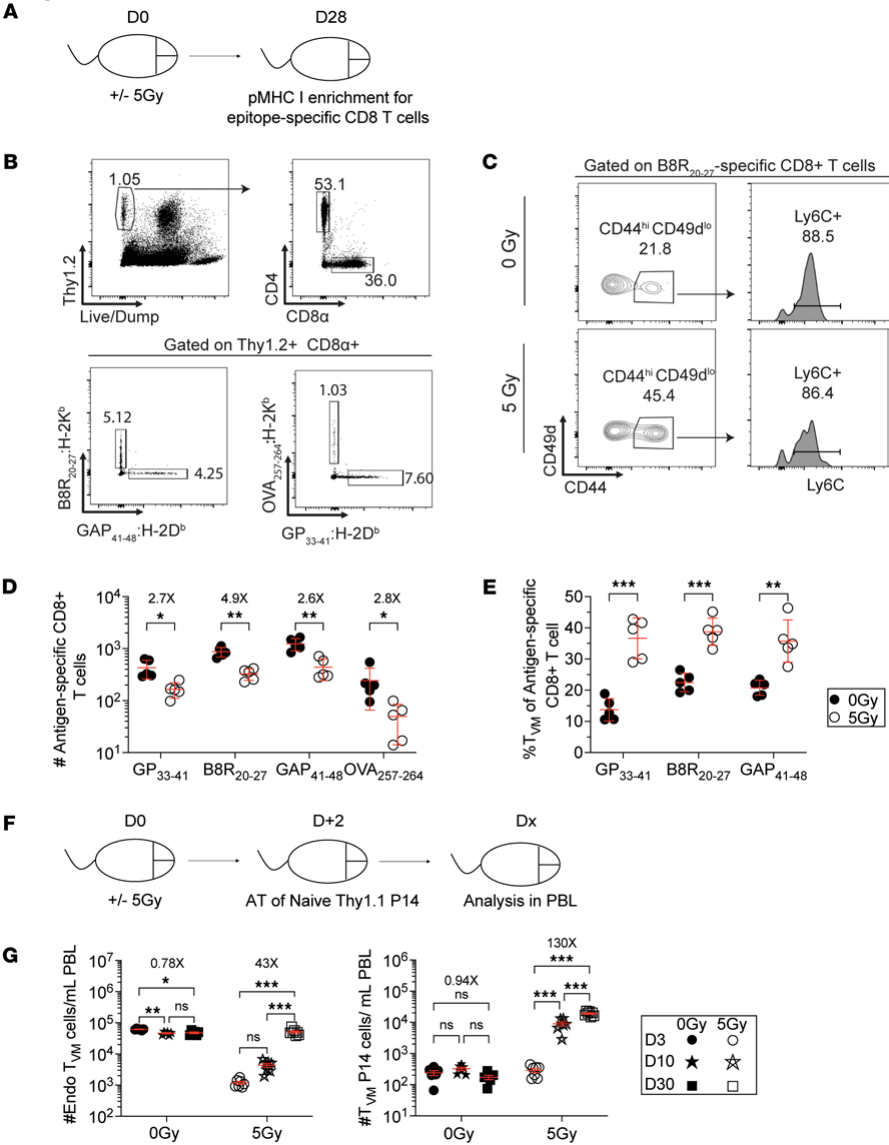
Acquisition of virtual memory phenotype by a fraction of proliferating T_N_ cells after WBI. (**A**) Experimental design: Thy1.2^+^ hosts were subjected to 0 Gy or 5 Gy dose of WBI. At 28 days later, the number and phenotype of Ag-specific CD8^+^ T cells specific for GP_33-41_, B8R_20-27_, GAP_41-48_, and OVA_257-264_ were determined using pMHC class I tetramer-based enrichment from spleens. (**B**) Representative flow plots showing gating strategy used to detect Ag-specific CD8^+^ T cell precursors and (**C**) virtual memory phenotype (CD44^hi^CD49^lo^Ly6C^+^) from Ag-specific CD8^+^ T cell precursors in 0 Gy and 5 Gy mice. (**D**) Absolute number and (**E**) frequency of virtual memory phenotype of D^b^-restricted GP_33-41_– and GAP50_40-48_–specific and K^b^-restricted B8R_20-27_– and OVA_257-264_–specific CD8^+^ T cells. (**F**) Experimental design: 7.5 × 10^5^ naive P14 CD8^+^ T cells were adoptively transferred to Thy1.2^+^ naive hosts that had undergone 0 Gy or 5 Gy WBI 2 days prior. Longitudinal blood analysis was performed at indicated time points. (**G**) Number of endogenous (left) and P14 (right) virtual memory (T_VM_) CD8^+^ T cells in 0 Gy and 5 Gy hosts at indicated time points after WBI. Data in **A**–**E** are from a single experiment, and **F** and **G** are representative of 2 independent experiments with *n* = 5–8 mice per group in each experiment. Statistical significance was determined by unpaired multiple *t* tests with Holm-Šídák multiple-comparison post hoc test or 2-way ANOVA with Bonferroni’s multiple comparisons post hoc test using GraphPad Prism. Graphs show the mean ± SEM with each symbol representing 1 mouse. Individual *P* values are noted on respective graphs or are summarized as follows: *P < 0.05, **P < 0.01, ***P < 0.001.

Our data in Figures 1 and 2 show the reconstitution of the bulk T_N_ population was concomitant with reconstitution and transient overrepresentation of the T_VM_ population. This observation prompted us to explore the potential for distinct changes within individual Ag-specific CD8^+^ T cell populations with respect to their numerical recovery and phenotypic change following WBI. Using a p:MHC I tetramer-based enrichment method (31, 54), we analyzed the precursor frequency and phenotype of 4 endogenous CD8^+^ T populations specific for ovalbumin (OVA_257-264_), LCMV-derived GP_33-41_, vaccinia virus-derived B8R_20-27_, and *Plasmodium*-derived GAP50_40-48_ epitopes in SPF hosts that underwent 0 or 5 Gy dose of WBI 28 days prior (Figure 6, A–C). Precursor numbers in 0 Gy hosts were consistent with previous analyses that reported various population sizes for each Ag-specific population with GAP50_40-48_–specific CD8^+^ T cells constituting the largest pool of Ag-specific cells followed by B8R_20-27_–, GP_33-41_–, and OVA_257-264_–specific repertoires (55, 56) (Figure 6D). Importantly, all 4 populations from 5 Gy mice showed a similar drop in population size compared to the 0 Gy mice. Additionally, phenotypic analysis of the populations with enough recovered cells (3 out of 4) showed an increased fraction of cells with T_VM_ phenotype, suggesting that following WBI, T_N_ cells can acquire a T_VM_ phenotype independent of their Ag specificity (Figure 6E). To formally show the capacity of T_N_ cells to give rise to T_VM_ progeny following WBI, we analyzed the number of P14 cells with T_VM_ phenotype following transfer of naive P14 cells 2 days after 0 or 5 Gy WBI (Figure 6F). In 5 Gy recipients, endogenous T_VM_ cells and P14 cells with a T_VM_ phenotype gradually increased in number over time, whereas their levels remained stable in 0 Gy recipients (Figure 6G). These data suggest that radiation-inexperienced T_N_ cells can acquire a T_VM_ phenotype following RIL.

**Figure 7:**
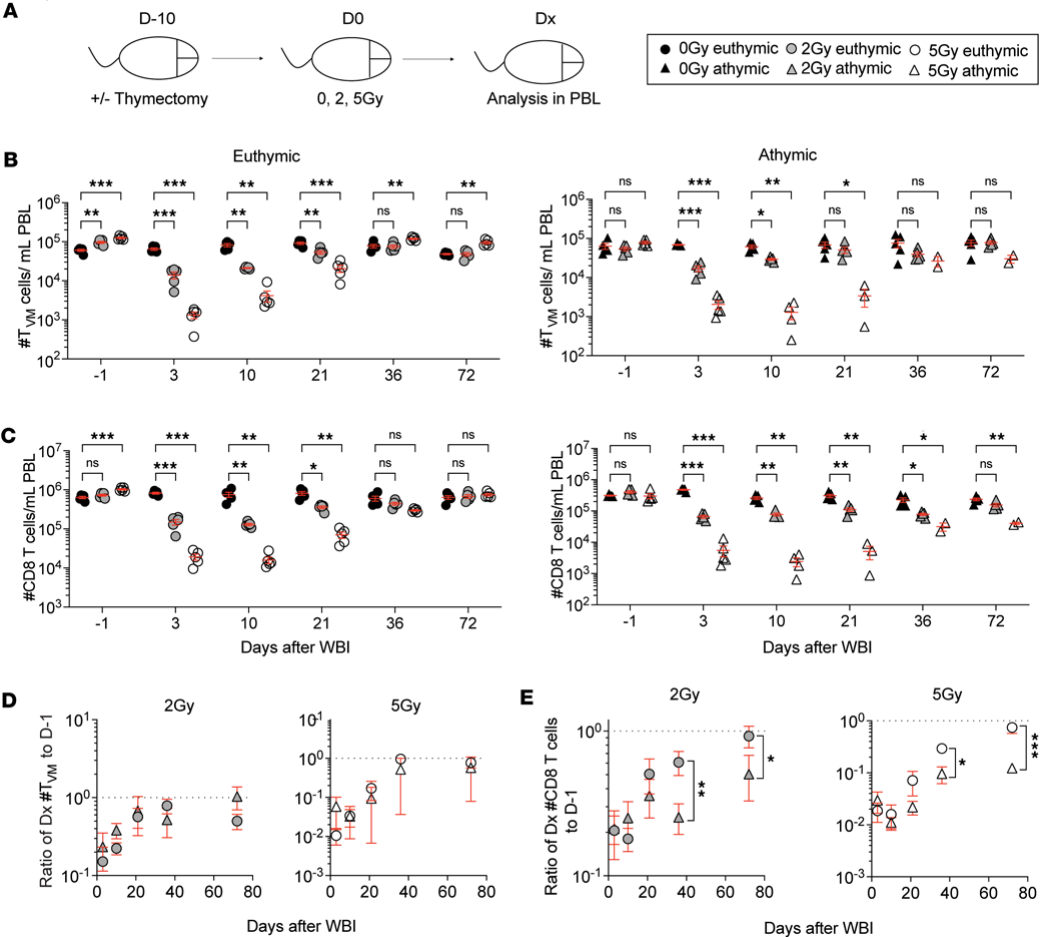
Repopulation of the virtual memory subset can occur in the absence of thymus. (**A**) Experimental design: same as Figure 3A. (**B**) Number of virtual memory (T_VM_) CD8^+^ T cells in blood of euthymic (left) and athymic mice (right) at indicated time points. (**C**) Number of total CD8^+^ T cells in blood of euthymic (left) and athymic mice (right) at indicated time points. Number of T_VM_ (**D**) and total CD8^+^ T cells (**E**) at each indicated time point was divided to the pre-WBI (D-1) number from the same mouse, and the mean ± SD of this ratio was plotted for 2 Gy (left) and 5 Gy (right) euthymic and athymic mice. Data in **A**–**E** are representative of 2 independent experiments with *n* = 2–5 mice per group in each experiment. Statistical significance was determined by 2-way ANOVA with Bonferroni’s multiple comparisons post hoc test. Graphs in **B** and **C** show the mean ± SEM with each symbol representing 1 mouse. Graphs in **D** and **E** show the mean ± SD. Individual *P* values are noted on respective graphs or are summarized as follows: *P < 0.05, **P < 0.01, ***P < 0.001.

Given the capacity of radiation-inexperienced T_N_ and T_VM_ cells to generate T_VM_ cells, we last asked whether T_N_ and T_VM_ cells that survived IR exposure can give rise to more T_VM_ cells following WBI (Figure 7A). To this end, we tracked the number of T_VM_ cells in PBL after euthymic and athymic mice were exposed to 0, 2, and 5 Gy doses of WBI. Interestingly, both euthymic and athymic mice were able to reconstitute their T_VM_ compartment to pre-WBI numbers (Figure 7, B and D), which was in sharp contrast with the diminished repopulation of T_N_ cells in athymic mice (Figure 7, B and D). Although the gradual repopulation of T_VM_ cells contributed to an increase in total CD8^+^ T cell numbers of both euthymic and athymic mice (Figure 7C), this was not sufficient to fully restore the CD8^+^ T cell numbers to the pre-WBI baseline in athymic mice (Figure 7E). These data collectively suggest that T_VM_ cells can arise from radiation-experienced T_N_ and T_VM_ cells.

## Discussion

Patients who receive high doses of IR as part of their solid tumor treatment or prior to hematopoietic stem cell transplant often experience a sustained lymphopenia that can vary greatly in duration (57). In addition to quantitative recovery, functional reconstitution of T cells also depends on balanced recovery of different subsets of T cells and TCR diversity (35). While T cell repopulation following WBI has been attributed to both HP of IR-surviving cells and T cell neogenesis in thymus, here we investigated the contribution of each mechanism to the reconstitution of Ag-inexperienced CD8^+^ T cells. We also investigated the short- and long-term effects of sublethal WBI on the phenotype and diversity of Ag-inexperienced CD8^+^ T cells. Our data support the notion that following IR-induced global loss of both T_N_ and T_VM_ cells, gradual numerical recovery of both subsets occurs, with the T_VM_ subset temporarily being enriched during early stages of lymphocyte recovery. While thymopoiesis is critical for efficient remission of T_N_ cells, the post-recovery T_N_ compartment may not fully recapitulate pre-WBI TCR diversity. Nevertheless, the T_N_ subset is never restored in the absence of the thymus, and only delayed T_VM_ recovery contributes to partial numerical recovery of the CD8^+^ T cell compartment.

We have previously demonstrated the inability of circulating and tissue-resident T_MEM_ cells to numerically increase following WBI (41, 58). Similarly, our results indicated the number of T_N_ cells that survived IR exposure remain low in euthymic mice, suggesting the contribution of such cells to the recovering T_N_ and T_VM_ subset is limited. Therefore, after WBI thymic neogenesis is required for effective regeneration of the T_N_ subset while differentiation of some of the newly generated thymic emigrants or mature T_N_ cells to a T_VM_ phenotype restores the T_VM_ subset in euthymic mice. Interestingly, athymic SPF mice exhibited a delayed but complete numerical recovery of the T_VM_ subset after WBI. Although the numerical expansion of IR-experienced T_N_ (or T_VM_) cells in athymic mice may look contradictory to the limited proliferative capacity of IR-experienced T_N_ cells in euthymic mice, one could postulate the new thymic emigrants that enter the lymphopenic environment after WBI outcompete the IR-experienced CD8^+^ T cells in sensing signals that drive HP and dominate the reconstitution of T_N_ and T_VM_ compartments. Therefore, it is only in the instance of thymic absence that IR-experienced CD8^+^ T cells receive adequate levels of homeostatic stimuli to substantially proliferate. Whether IR-experienced T_N_ and/or T_VM_ cells contribute to the T_VM_ subset recovery in athymic mice remains to be determined.

Although thymus-derived CD8^+^ T cells are instrumental in reconstituting the T_N_ subset after WBI, phenotypic and TCR clonotype composition of T_N_ cells is altered after numerical recovery. Altered TCR diversity among T_N_ cells may stem from differential capacity of the newly generated T_N_ cells to undergo HP in the lymphopenic environment after WBI, leading to changes to the composition of the TCR repertoire. Additionally, we noted an increase in the population of Ly6C^+^ T_N_ cells for up to 60 days in 5 Gy hosts. Ly6C expression is induced by type I interferon (IFN) signaling, which is upregulated shortly after many intracellular infections, microbial exposure through cohousing, and polymicrobial sepsis (53, 59). We speculate the IR-induced increase in type I IFN (60, 61) in conjunction with increased TCR contact with self-pMHC because of prolonged lymphopenia promotes Ly6C upregulation on the newly emerging T_N_ cells. Additionally, Ly6C^+^ T_N_ cells proliferate vigorously in the lymphopenic environment, which can further lead to an enrichment of Ly6C^+^ T_N_ cells after WBI. Ly6C^+^ T_N_ cells are endowed with a greater effector and memory formation capacity and bystander activation than Ly6C^–^ T_N_ cells (52, 59). This enrichment may allow for a more robust effector response in hosts that are still recovering from RIL and hence confer advantage in combating new and recurring pathogens.

We provide evidence that T_VM_ phenotype acquisition by Ag-specific CD8^+^ T cells occurs independent of their specificity in euthymic mice. The rapid recovery of the T_VM_ subset in irradiated euthymic mice is reminiscent of emergence of phenotypically similar HP memory CD8^+^ T cells that arise after vigorous expansion and memory marker acquisition of T_N_ cells upon adoptive transfer to sublethally irradiated mice (62–64). While T_VM_ and HP memory CD8^+^ T cells both share memory markers, whether both represent the same population is not well understood, as they arise under different homeostatic circumstances. It is crucial to investigate whether the propensity of after WBI T_VM_ cells in differentiating to Ag-specific effector and true memory cells is any different than the T_VM_ cells in nonirradiated mice. Additionally, as IR exposure impairs the cognate Ag-derived effector function of T_MEM_ cells, it is plausible that IR-experienced T_N_ and T_VM_ cells have reduced per-cell capacity to give rise to Ag-specific effector and true memory cells, but this hypothesis remains to be investigated.

Human T_VM_ cells have been identified as CD45RA^+^CCR7^–^CD8^+^ T cells that express KIR/NKG2A (28, 65). Similar to the pool of T_MEM_ cells, T_VM_ cells accumulate with age while the thymic output and T_N_ pool size progressively diminish (28, 66, 67). An important finding of the current study is the elevated proportions of T_VM_ cells within the CD8^+^ T cell pool in mice with history of pathogen exposure and athymic mice following WBI and subsequent CD8^+^ T cell reconstitution. Unlike in SPF mice, the overrepresentation of T_VM_ cells in LCMV-immune mice was likely due to the WBI-mediated permanent loss of approximately 80% of pathogen-specific circulatory memory CD8^+^ T cells while Ag-inexperienced CD8^+^ T cells gradually repopulated and established the pre-WBI numbers. In addition, only the T_VM_ subset was fully restored in athymic SPF mice while the number of T_N_ cells stalled. Using classic gating (CD45RA vs. CCR7), human T_VM_ cells fall within a larger subset of CD8^+^ T cells termed as terminal effector memory CD8^+^ T cells that expand in patients recovering from allogeneic hematopoietic stem cell transplant (68). Our findings may suggest that in individuals recovering from an episode of severe lymphopenia, the T_VM_ subset may constitute a greater fraction of total CD8^+^ T cells than previously appreciated and may warrant further investigation.

Differences in murine and human lymphocyte response to IR and RIL may limit the extent to which the findings of this study can be extended. The relatively expeditious numerical recovery of lymphocytes in adult mice following WBI-mediated or focal brain irradiation–induced RIL does not recapitulate the prolonged RIL episodes that are often seen in patients who underwent radiotherapy (69). In addition, previous studies have pointed to differential gene expression patterns in mouse and human blood cells following exposure to high doses of IR, which may influence the fate of IR-experienced CD8^+^ T cells in both species (70). Notably, blood cells from hematopoietically humanized mice display IR-induced gene expression profiles more similar to those of human WBI blood samples than those of murine blood cells (70). Hence, it would be of interest to investigate the dynamic changes to the number and phenotype of CD8^+^ T cell subsets in hematopoietically humanized mice following IR exposure.

In summary, this study provides an overview of the dynamic changes to the Ag-inexperienced CD8^+^ T cell subset following WBI. Our findings emphasize the indisputable role of thymus in T_N_ regeneration while T_VM_ recovery can be achieved in the absence of thymopoiesis. While we documented lasting changes to the subset representation and TCR diversity of T_N_ cells after WBI, future studies on functional implications of such alterations will inform therapeutic approaches to address lymphopenia and the immune incompetence associated with it.

## Methods

### Sex as a biological variable.

Experiments were conducted using both male and female mice, with no differences observed in the kinetic of loss, recovery, and phenotypic changes to the bulk Ag-inexperienced CD8^+^ T cells following WBI. Thereafter, our experimental designs involving adoptive transfer of transgenic CD8^+^ T cells, thymectomized mice, and MHC class I tetramer-based enrichment utilized female mice, with the findings expected to be relevant for all sexes.

### Mice, infections, and memory CD8^+^ T cell generation.

Inbred C57BL/6 (Thy 1.2/1.2) mice were purchased from Charles River and maintained in the animal facilities at the University of Iowa at the appropriate biosafety level. Thymectomized C57BL/6 mice were purchased from Jackson Laboratory. P14 TCR-transgenic mice (Thy1.1/1.1) were bred and maintained at the University of Iowa (Iowa City, Iowa, USA).

To generate memory CD8^+^ T cells, 10^4^ naive P14 TCR-Tg CD8^+^ T cells were adoptively transferred into C57BL/6 mice, followed by infection with 2 × 10^5^ PFU of LCMV-Armstrong by i.p. injection a day later. In experiments with adoptive transfer of naive P14 CD8^+^ T cells but no subsequent LCMV infection, 0.75 × 10^6^ to 1.0 × 10^6^ naive cells were transferred.

### Irradiation.

WBI was performed by placing the mice in a 225 kv rotating x-ray tube (Small Animal Radiation Research platform; Xstrahl) available at the Radiation Core at the University of Iowa. Radiation doses of 2 Gy and 5 Gy were used in this study. Mock-treated mice were placed into the same mouse pie cages as IR-treated mice were and remained there for the length of a 5 Gy exposure without WBI exposure.

### Cell isolation.

Peripheral blood was collected by retro-orbital bleeding. Single-cell suspensions from spleen and lymph nodes were generated after mashing tissue through a 70 μm cell strainer (Corning) without enzymatic digestion. ACK lysis buffer was used for red blood cell lysis from peripheral blood and spleen samples.

### Cell staining and flow cytometry.

Single-cell suspensions were incubated with a cocktail of fluorescently labeled mAbs for 30 minutes at 4°C. These mAbs included: CD8a 53-6.7, BioLegend), CD11a (M17/4, BioLegend), Thy1.1 (OX7, BioLegend), CD49d (R1-2, BioLegend), CD62L (MEL14, BioLegend), CD44 (IM7, BioLegend), CD4 (GK1.5, BioLegend), CD19 (1D3, BioLegend), Vβ2 (B20.6, BioLegend), Vβ3 (KJ25, BD), Vβ5.1/5.2 (MR9-4, BioLegend), Vβ6 (RR4-7, eBioscience), Vβ8.1/8.2 (KJ16-133, eBioscience), Vβ8.3 (1B3.3, BioLegend), Vβ10b (B21.5, BioLegend), Vβ11 (KT11, BioLegend), Vβ13 (MR12-3, eBioscience), Vβ14 (14-2, BD), Ly6C (HK1.4, BioLegend) CD122 (5H4, BioLegend), and CD127 (eBioSB/199, eBioscience). Samples were then fixed with Cytofix (BD Biosciences) at 4°C for 15 minutes. To stain for Ki-67 (B56, BD Biosciences), Foxp3/Transcription Factor Staining Buffer reagents (eBioscience) and protocol were used. Flow cytometry data were acquired using an LSRFortessa (BD Biosciences) and analyzed using FlowJo software v.10 (FlowJo LLC) using FlowSOM and tSNE plug-ins.

### Enrichment and characterization of endogenous Ag-specific naive CD8^+^ T cells.

Tetramer-based enrichment protocol (31, 71) using GP_33-41_ and GAP50_40-48_ containing D^b^ MHC class I tetramer and OVA_257-264_ and B8R_20-27_ containing K^b^ MHC class I tetramers was used. In brief, single-cell suspension from splenocytes was stained with PE- and APC-conjugated pMHC I tetramers in 300 μL tetramer staining buffer (PBS containing 5% FBS, 2 mM EDTA, 1:50 normal mouse serum, and 1:100 anti-CD16/32 mAb). The cells were incubated in the dark at room temperature for 1 hour, followed by a wash in 10 mL ice-cold FACS Buffer. The tetramer-stained cells were then resuspended in 300 μL FACS Buffer, mixed with 25 μL of anti-PE and anti-APC mAb-conjugated magnetic microbeads (STEMCELL Technologies), and incubated in the dark on ice for 30 minutes. The cells were washed, resuspended in 3 mL cold FACS Buffer, and passed through an EasySep Magnet (STEMCELL Technologies) to yield the enriched tetramer-positive population. The resulting enriched fractions were stained with a cocktail of fluorochrome-labeled mAbs: Thy1.2 (53–2.1, eBioscience), CD4, CD8, CD44, CD49d, Ly6C, and “dump/live” (CD11b [M1/70, eBioscience], CD11c [N418, eBioscience], B220 [RA3-6B2, eBioscience], F4/80 [BM8, eBioscience], Live/Dead [eBioscience]). Cell numbers for each sample were determined using AccuCheck Counting Beads (Invitrogen). Samples were then analyzed using a Fortessa flow cytometer (BD Biosciences) and FlowJo software (TreeStar).

### Statistics.

All statistical analyses were performed using GraphPad Prism (v10.0). When indicated, 2-tailed unpaired Student’s *t* tests were performed when comparing 2 independent groups and 2-way ANOVA with Bonferroni’s multiple comparisons test when comparing more than 2 groups for more than 1 variable. *P* values are indicated in individual figures or in figure legends. *P* < 0.05 was considered significant.

### Study approval.

Experimental procedures using mice were approved by the University of Iowa Animal Care and Use Committee under protocol numbers 2121915.

### Data availability.

All data associated with this study can be found in the main text or the supplement. Values for all data points in graphs are reported in the Supporting Data Values files.

## Author contributions

MH, JTH, and VPB conceived and the designed the study. MH, SKK, and WS conducted experiments and analyzed data. MH and VPB wrote the original draft, and SKK, VPB, TSG, and JTH reviewed and edited the manuscript. TSG, JTH, and VPB acquired funding and supervised the project.

## Supplementary Material

Supplemental data

Supporting data values

## Version 1 08/05/2025

In-Press Preview

## Version 2 09/23/2025

Electronic publication
