# Supplementary material for: Delineating the short- and long-term impact of ionizing radiation on antigen-inexperienced CD8+ T cell subsets
Source: JCI Insight. 2025 Aug 5;10(18):e194201. doi: 10.1172/jci.insight.194201 (PMC12487864; doi:10.1172/jci.insight.194201)
Supplement: Supplemental data [file jciinsight-10-194201-s043.pdf]

Supplemental Figure 1

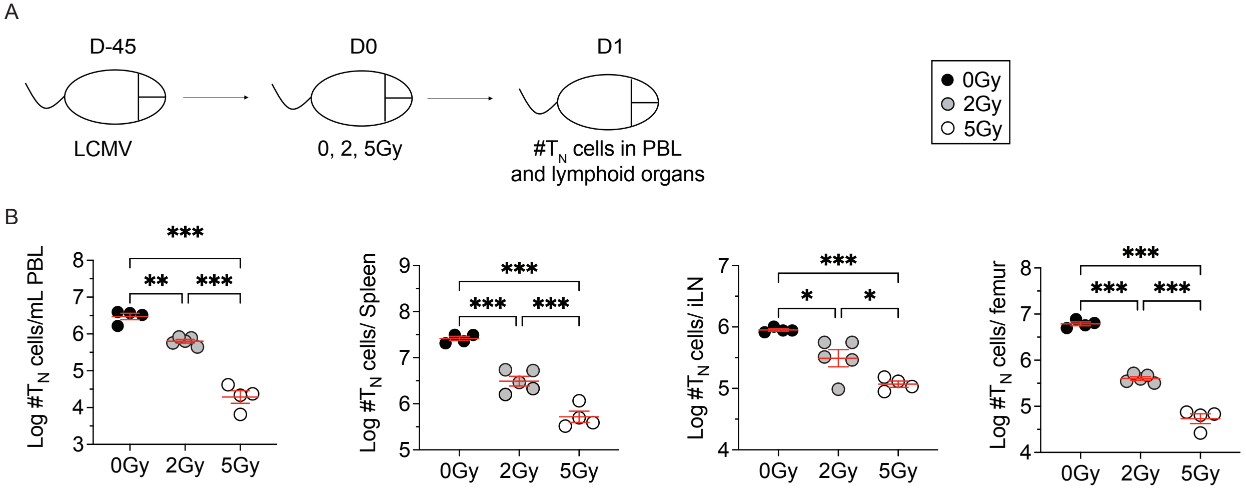

Supp. Fig 1. Dose-dependent loss of naïve $CD8^+$ T cells following WBI. (A) Experimental design: 10^4 naive Thy1.1$^+$ P14 $CD8^+$ T cells were adoptively transferred into Thy1.2$^+$ naïve hosts, followed by LCMV-Armstrong infection to generate memory P14 CD8 T cells. 45 days later, the memory P14 chimeric mice were either exposed to mock (0Gy), 2Gy, or 5Gy WBI. Analysis was performed on the indicated organs one day after WBI. (B) Number of naïve (T_N) $CD8^+$ T cells from 0Gy, 2Gy and 5Gy mice in peripheral blood (PBL), spleen, inguinal lymph node (iLN), and femur. Data are representative of 2 independent experiments with n=4-5 mice per group in each experiment. Statistical significance was determined by one-way ANOVA with Bonferroni's multiple comparisons post-hoc test using GraphPad Prism. Graphs show the mean ± s.e.m. with each symbol representing one mouse. Individual *P* values are noted on respective graphs or are summarized as follows: $*P < 0.05$, $**P < 0.01$, $***P < 0.001$.

Supplemental Figure 2

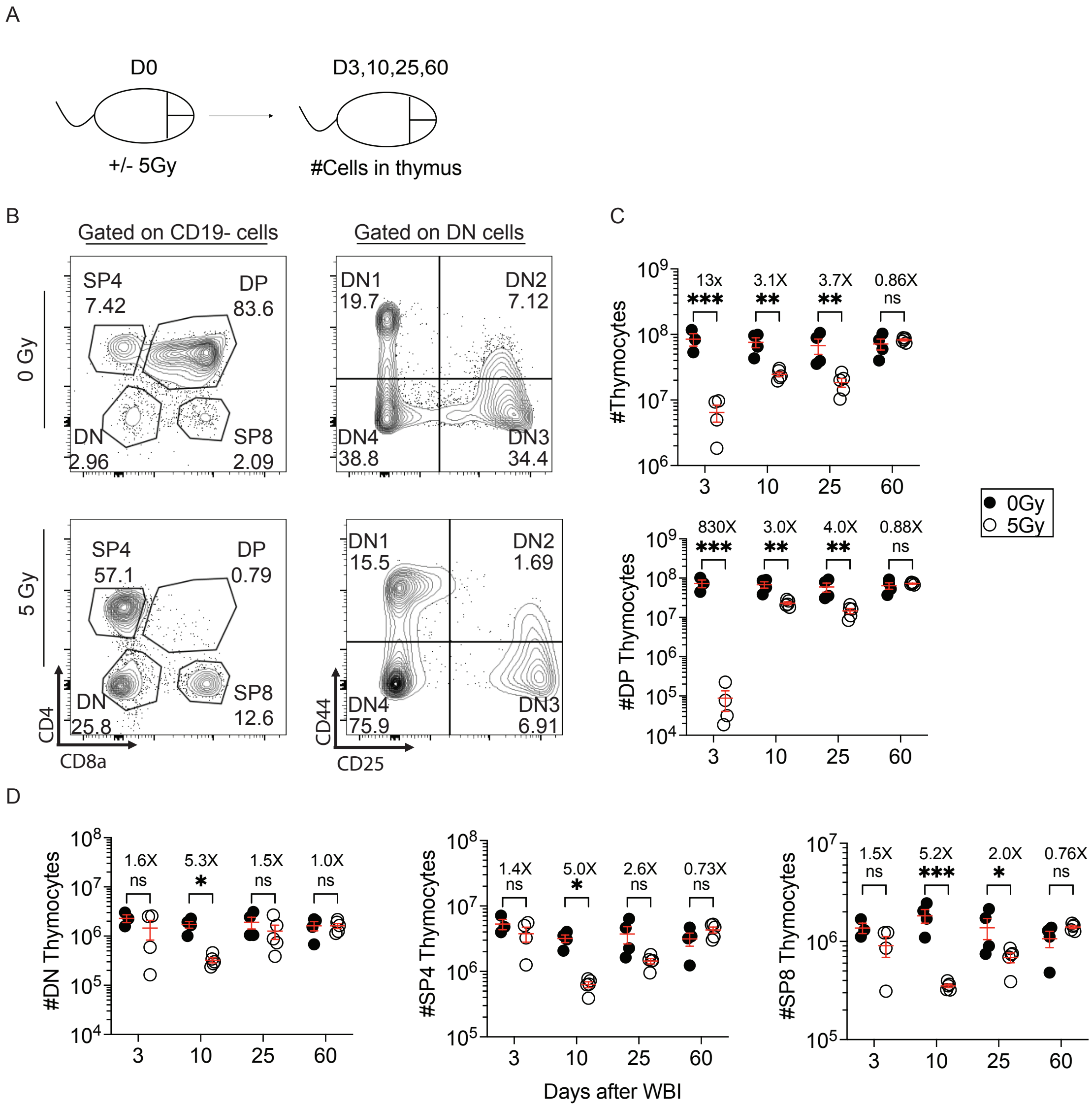

Supp. Fig 2. Thymocyte numerical recovery precedes naïve CD8+ T cell recovery. (A) Experimental design: SPF mice were subjected to either 0Gy or 5Gy WBI. At indicated timepoints, mice were euthanized for cellular analysis in thymus. (B) Representative flow plots of double positive (DP), single positive CD4+ (SP4) and CD8+ (SP8) and different subsets of double negative (DN) thymocytes. (C and D) Number of total thymocytes, DP, DN, SP4 and SP8 thymocytes at indicated timepoints post-WBI. Data in (A-D) are representative of 2 independent experiments with n=4-5 mice per group in each experiment. Statistical significance was determined by two-way ANOVA with Bonferroni's multiple comparisons post-hoc test using GraphPad Prism. Graphs show the mean ± s.e.m. with each symbol representing one mouse. Individual P values are noted on respective graphs or are summarized as follows: *$P < 0.05$, **$P < 0.01$, ***$P < 0.001$.

Supplemental Figure 3

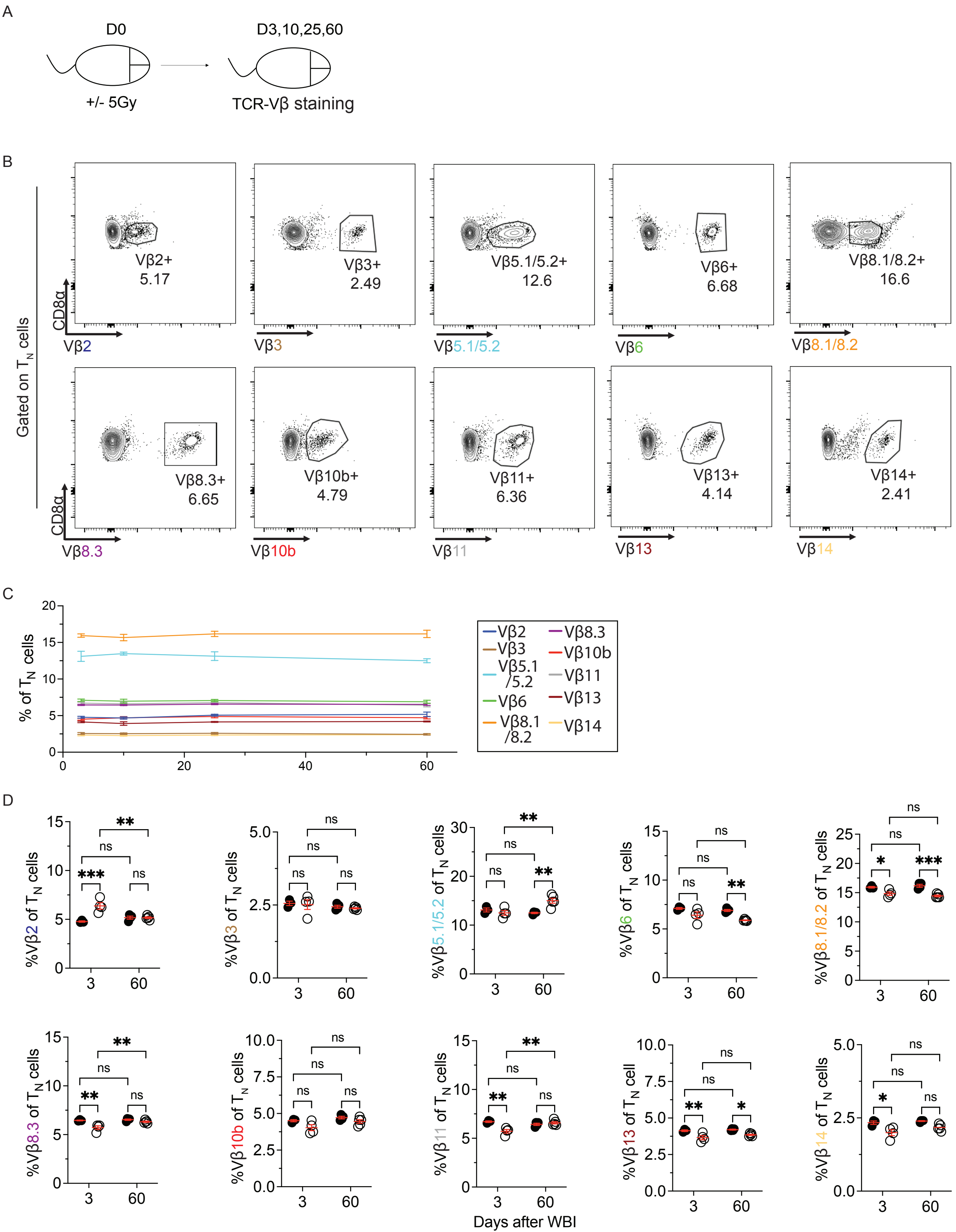

Supp. Fig 3. Altered clonal diversity of naïve CD8$^+$ T subset after recovery from WBI. (A) Experimental design: SPF mice were subjected to either 0Gy or 5Gy WBI. At indicated timepoints, mice were euthanized for cellular analysis in spleen. (B) Representative flow plots of TCR Vβ-specific naïve (T_N) CD8$^+$ T cells (C) and their representation in 0Gy mice over time. (D) comparison of TCR Vβ-specific T_N cells between 0Gy and 5Gy mice at D3 and D60 post-WBI. Data in (A-D) are representative of 2 independent experiments with n=4-5 mice per group in each experiment. Statistical significance was determined by two-way ANOVA with Bonferroni's multiple comparisons post-hoc test using GraphPad Prism. Graphs show the mean ± s.e.m. with each symbol representing one mouse. Individual *P* values are noted on respective graphs or are summarized as follows: $^*P < 0.05$, $^{**}P < 0.01$, $^{***}P < 0.001$.
